# Prognostic impact and predictors of persistent renal dysfunction in acute kidney injury after percutaneous coronary intervention for acute myocardial infarction

**DOI:** 10.1038/s41598-024-56929-y

**Published:** 2024-03-15

**Authors:** Takuya Nakamura, Makoto Watanabe, Junichi Sugiura, Atsushi Kyodo, Saki Nobuta, Kazutaka Nogi, Yasuki Nakada, Satomi Ishihara, Yukihiro Hashimoto, Hitoshi Nakagawa, Tomoya Ueda, Ayako Seno, Taku Nishida, Kenji Onoue, Shungo Hikoso

**Affiliations:** Department of Cardiovascular Medicine, Nara Medical University, 840 Shijo-cho, Kashihara, Nara 634-8522 Japan

**Keywords:** Cardiology, Nephrology

## Abstract

This study aimed to evaluate the prognostic impact and predictors of persistent renal dysfunction in acute kidney injury (AKI) after an emergency percutaneous coronary intervention (PCI) for acute myocardial infarction (AMI). A total of 877 patients who underwent emergency PCI for AMI were examined. AKI was defined as serum creatinine (SCr) ≥ 0.3 mg/dL or ≥ 50% from baseline within 48 h after PCI. Persistent AKI was defined as residual impairment of SCr ≥ 0.3 mg/dL or ≥ 50% from baseline 1 month after the procedure. The primary outcome was the composite endpoints of death, myocardial infarction, hospitalization for heart failure, stroke, and dialysis. AKI and persistent AKI were observed in 82 (9.4%) and 25 (2.9%) patients, respectively. Multivariate Cox proportional hazards analysis demonstrated that persistent AKI, but not transient AKI, was an independent predictor of primary outcome (hazard ratio, 4.99; 95% confidence interval, 2.30–10.8; P < 0.001). Age > 75 years, left ventricular ejection fraction < 40%, a high maximum creatinine phosphokinase MB level, and bleeding after PCI were independently associated with persistent AKI. Persistent AKI was independently associated with worse clinical outcomes in patients who underwent emergency PCI for AMI. Advanced age, poor cardiac function, large myocardial necrosis, and bleeding were predictors of persistent AKI.

## Introduction

Acute kidney injury (AKI) is a frequent complication in patients with acute myocardial infarction (AMI) undergoing primary percutaneous intervention (PCI) compared to those undergoing elective PCI^1,2^ and is known to be an independent risk factor for increased long-term mortality and worse clinical outcomes^3,4^.

The cause of AKI in patients with AMI is multifactorial because AKI develops not only because of the large amount of contrast medium exposure during PCI but also because of hemodynamic instability, renal hypoperfusion following impaired cardiac output, and systemic inflammatory response due to ischemic injury and myocardial necrosis^5^.

Most AKI cases after PCI are transient and improve within 2 weeks, whereas some patients have persistent renal dysfunction after the development of AKI^6^. However, the impact of persistent or transient renal dysfunction on worse clinical outcomes, including major adverse cardiovascular events (MACEs) and dialysis, and predictors of persistent renal dysfunction remain unclear in patients with AMI undergoing PCI.

This study aimed to investigate the impact of renal function reversibility following AKI on clinical outcomes and predictors of persistent renal dysfunction in patients with AMI undergoing emergency PCI.

## Methods

### Study design and population

**Figure 1:**
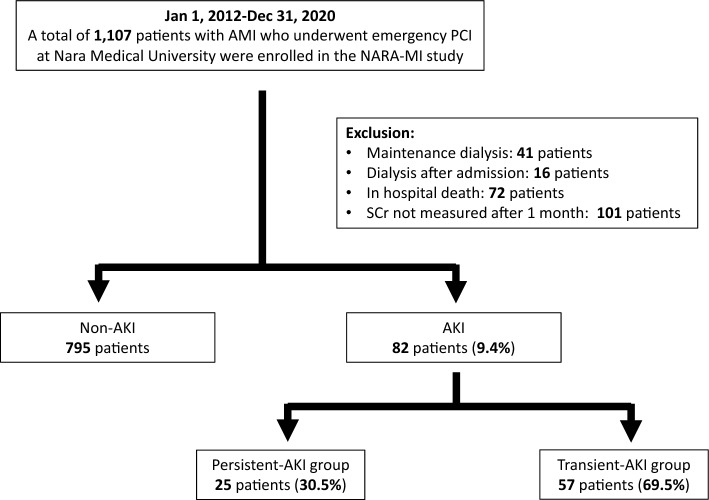
Study flow. *AKI* acute kidney injury, *AMI* acute myocardial infarction, *SCr* serum creatinine.

This was a single-center, retrospective, observational study. Patients with AMI who underwent emergency PCI at Nara Medical University Hospital between January 2012 and December 2020 were enrolled. The diagnoses of AMI included ST-elevation myocardial infarction (STEMI) and non-ST-elevation myocardial infarction (NSTEMI) within 48 h of AMI onset. STEMI was defined as continuous chest pain, ST-segment elevation in two contiguous leads or a new left bundle branch block on 12-lead electrocardiography, and elevated cardiac marker levels (creatine kinase-MB or troponin). NSTEMI was defined as ischemic symptoms in the absence of ST-segment elevation on electrocardiography with elevated cardiac marker levels^7^. Patients on previous chronic dialysis or who underwent hemodialysis after admission, died in hospital, or lacked data on serum creatinine (SCr) levels were excluded from the study. Figure 1 shows the enrollment and exclusion criteria and study flow. Primary PCI was performed using standard techniques and catheters via the femoral or radial approach according to the operator’s usual practice.

### Data collection and clinical definition

Baseline data including clinical characteristics, laboratory data, medication upon admission, and procedural data were obtained for all patients.

Baseline laboratory data included hemoglobin (Hb), SCr, HbA1c, and maximum creatine phosphokinase MB (Max CKMB) levels and estimated glomerular filtration rate (eGFR). Anemia was defined as Hb < 12 g/dL in males and < 11 g/dL in females. The eGFR was calculated as follows using the “Japanese Modification of Diet in Renal Disease study equation” published by the Japanese Society of Nephrology: eGFR (men) = 194 × serum creatinine − 1.094 × age − 0.287 and eGFR (women) = eGFR (men) × 0.739^8^. Procedural data included STEMI or NSTEMI, culprit vessel (left anterior descending artery, left circumflex artery, right coronary artery, or left main trunk), diseased vessels (one or multiple vessels), Killip class ≥ 3, approach site for PCI (femoral artery or not), multivessel PCI in hospital (one-time or staged strategy), volume of contrast media, thrombolysis in myocardial infarction flow grade (TIMI) score before and after PCI, use of mechanical circulatory support (intra-aortic balloon pumping or extracorporeal membrane oxygenation) during PCI, use of catecholamine, and perioperative major bleeding complication within 48 h after PCI, defined as Bleeding Academic Research Consortium (BARC)^9^ type 3 or greater. Echocardiography was performed to evaluate left ventricular ejection fraction (EF) within 1 week of the procedure. SCr levels were measured before the procedure (baseline), every day for the following 2 days, and 1 month after the procedure to identify patients without AKI (non-AKI), those with persistent AKI, and those with transient AKI. AKI was defined as an increase in SCr ≥ 0.3 mg/dL or ≥ 50% from baseline within 48 h^10^. Patients diagnosed with AKI were divided into those with persistent AKI and those with transient AKI. Persistent AKI was defined as residual impairment of SCr ≥ 0.3 mg/dL or ≥ 50% from baseline 1 month after procedure. Transient AKI was defined as recovery to SCr < 0.3 mg/dL and < 50% from baseline 1 month after the procedure. The Mehran score was calculated based on eight clinical and procedural variables: age > 75 years, hypotension, congestive heart failure, use of an intra-aortic balloon pump, serum creatinine level, diabetes, anemia, and volume of contrast, according to a previous report^11^.

### End points

The primary outcome of this study was a composite of MACEs, including death, myocardial infarction, hospitalization for heart failure, stroke, and initiation of maintenance dialysis. The secondary endpoints were MACEs, mortality, and dialysis. Clinical follow-up was conducted through outpatient visits or telephone interviews.

### Statistical analysis

The Shapiro–Wilk test was used to evaluate the distribution of continuous data. Normally distributed data are expressed as mean ± standard deviation (SD), and those with skewed distributions are expressed as median with interquartile range, whereas categorical variables are presented as counts and percentages. Categorical data were compared using the Pearson χ^2^ test. Continuous variables were compared using parametric one-way analysis of variance or the non-parametric Kruskal–Wallis test, based on the distribution of variables. The cumulative incidence of survival-free periods from clinical events was estimated using the Kaplan–Meier method. In the case of significant differences, pairwise post-hoc tests were performed with Bonferroni correction. A univariate Cox proportional hazards model was used to identify variables associated with the primary outcomes in the present study. Two multivariate Cox proportional hazards models were used to identify independent predictors of the primary outcome, including 12 variables with P < 0.05 in the univariate model. One model included post PCI TIMI score < 3, and the other included mechanical circulatory support. Univariate logistic regression analysis was used to identify the significant clinical factors associated with the development of AKI and persistent AKI. A multivariate logistic regression model was used to identify independent predictors of persistent AKI, which included variables with P < 0.05, in the univariate model. A P < 0.05 was considered statistically significant. All statistical analyses were performed using JMP software version 16 (SAS Institute JAPAN Corporation, Roppongi, Tokyo).

### Ethics statement

This study was approved by the Ethics Committee of Nara Medical University (Reference no. 2162) and complied with the Declaration of Helsinki’s Ethical Principles for Medical Research Involving Human Subjects. Informed consent was obtained in the form of an opt-out option on the Department of Cardiovascular Medicine, Nara Medical University website.

## Results

### Patient characteristics

Of the 1107 consecutive patients, 230 were excluded (41 were on maintenance hemodialysis, 16 were undergoing temporary hemodialysis in the hospital, 72 were in-hospital deaths, and 101 lacked data on SCr). Finally, 877 consecutive patients were included in the study, and AKI was present in 82 (9.4%). Of the AKI cases, persistent and transient AKIs were present in 25 (30.5%) and 57 (69.5%) patients, respectively (Fig. 1).

Table 1 shows a comparison of the baseline clinical characteristics among the three groups. The persistent AKI group was older and more frequently had anemia and a lower left ventricular ejection fraction (EF < 40%) than the non-AKI group. The transient AKI group was older; had a higher prevalence of diabetes, lower baseline eGFR levels, and lower EF; and more frequent use of angiotensin II receptor blocker (ARB), angiotensin-converting enzyme inhibitors (ACE-Is), and angiotensin receptor neprilysin inhibitor (ARNI) than the non-AKI group. There were no significant differences in the baseline clinical characteristics between the persistent and transient AKI groups. Table 2 compares the baseline lesion and procedural characteristics among the three groups. Transient AKI group had a higher incidence of severe myocardial infarction with Killip ≥ 3 and a higher contrast volume/eGFR ratio compared with non-AKI group. The incidence of BARC type 3 or greater bleeding complication was significantly higher in the persistent and the transient AKI groups compared to the non-AKI group. The Mehran risk score was significantly higher in the persistent and transient AKI groups than in the non-AKI group. There were no significant differences in baseline lesion and procedural characteristics between the persistent and transient AKI groups.

**Table 1:** Baseline clinical characteristics.

	Persistent AKI	Transient AKI	No AKI	P value
Age, year	77 (70.5–85)*	73 (66.5–83.5)^†^	69 (60–76)*^,†^	**< 0.001**
Age > 75 years	14 (56.0)*	25 (43.9)^†^	216 (27.2)*^,†^	**0.0003**
Male gender	16 (64.0)	44 (77.2)	618 (77.3)	0.27
Diabetes mellitus	10 (40.0)	31 (54.4)^†^	270 (34.0)^†^	**0.007**
Dyslipidemia	11 (44.0)	28 (50.0)	447 (56.2)	0.33
Hypertension	17 (68.0)	42 (73.7)	519 (65.3)	0.42
History of MI	1 (4.0)	5 (8.8)	44 (5.5)	0.56
Stroke	4 (16.0)	6 (10.5)	55 (6.9)	0.15
PAD	2 (8.0)	4 (7.0)	17 (2.1)	**0.02**
Hb, g/dL	13.2 (12.2–14.8)	13.3 (11.1–15.2)^†^	14.2 (13.0–15.2)^†^	**0.0019**
Anemia	14 (56.0)*	29 (50.9)^†^	204 (25.7)*^,†^	**< 0.0001**
SCr, mg/dL	1.00 (0.67–1.33)	1.02 (0.74–1.37)^†^	0.86 (0.72–1.02)^†^	**0.0019**
eGFR, mL/min/1.73 m^2^	52.2 (40.2–74.0)	53.1 (37.8–77.5)^†^	66.6 (53.5–78.4)^†^	**0.0004**
HbA1c	6.5 (5.7–7.2)	6.3 (5.8–7.1)	6.0 (5.7–6.8)	0.15
Max CKMB, IU/L	312 (155–481)	190 (77–378)	186 (88–345)	0.06
EF, %	46.0 (36.0–58.0)*	52.7 (42.8–66.1)	58.0 (50.0–64.0)*	**0.0002**
EF < 40%	7 (30.4)*	12 (23.1)^†^	52 (6.7)*^,†^	**< 0.0001**
Medication				
ARB, ACE-Is or ARNI	11 (44.0)	26 (45.6)^†^	230 (29.2)^†^	**0.016**
β-blocker	7 (28.0)	7 (12.5)	62 (8.0)	0.051
Statin	8 (32.0)	20 (35.1)	169 (21.5)	0.36

Unless otherwise indicated, the values are presented as medians (interquartile ranges) or n (%).

*MI* myocardial infarction, *PAD* peripheral artery disease, *Hb* hemoglobin, *SCr* serum creatinine, *eGFR* estimated glomerular filtration rate, *CKMB* creatine kinase MB, *EF* ejection fraction, *ARB* angiotensin II receptor blocker, *ACE-Is* angiotensin-converting enzyme inhibitors, *ARNI* angiotensin receptor neprilysin inhibitor.

*Persistent AKI vs. No AKI, P < 0.017 (Bonferroni correction). ^†^Transient AKI vs No AKI, P < 0.017 (Bonferroni correction).

Significant values are in bold.

**Table 2:** Baseline lesion and procedural characteristics.

	Persistent AKI 25 (2.9%)	Transient AKI 57 (6.5%)	No AKI 795 (90.6%)	P value
Clinical presentation at PCI				0.31
STEMI	22 (88.0)	43 (75.4)	651 (82.5)	
NSTEMI	3 (12.0)	14 (24.6)	138 (17.5)	
Culprit lesion				0.11
LAD	15 (60.0)	28 (50.0)	360 (45.3)	
RCA	6 (24.0)	18 (32.1)	319 (40.2)	
LCX	4 (16.0)	6 (10.7)	102 (12.9)	
LMT	0 (0)	4 (7.1)	13 (1.6)	
Multiple vessel disease	10 (40.0)	18 (31.6)	239 (30.1)	0.56
Multivessel PCI with one-time strategy	2 (8.0)	6 (10.5)	32 (4.0)	**0.05**
Multivessel PCI with staged strategy	6 (24.0)	12 (21.1)	193 (24.3)	0.86
Killip ≥ 3	5 (20.0)	12 (21.4)^†^	73 (9.3)^†^	**0.012**
Femoral approach	16 (64.0)	32 (57.1)	364 (45.8)	0.058
Contrast volume, mL	170 (128–219)	178 (121–232)	184 (142–228)	0.46
Contrast volume/eGFR	3.31 (2.40–4.43)	3.21 (2.56–4.57)^†^	2.79 (2.18–3.52)^†^	**0.0026**
Pre-PCI TIMI score < 3	23 (92.0)	53 (94.6)	705 (88.9)	0.31
Post-PCI TIMI score < 3	1 (4.0)	4 (7.1)	47 (5.9)	0.86
Mechanical circulatory assist devices, n (%)	3 (12.0)	14 (24.6)	105 (13.2)	0.08
Catecholamine use, n (%)	3 (12.0)	11 (19.3)	103 (13.2)	0.46
Bleeding BARC type ≥ 3	5 (20.0)*	9 (15.8)^†^	29 (3.7)*^,†^	**< 0.0001**
Mehran score	10.0 (6.0–14.0)*	11.5 (6.0–16.8)^†^	6.0 (3.0–11.0)*^,†^	**< 0.0001**

Unless otherwise indicated, the values are presented as medians (interquartile ranges) or n (%).

*PCI* percutaneous coronary intervention, *STEMI* ST-segment elevation myocardial infarction, *NSTEMI* non-ST-segment elevation myocardial infarction, *LAD* left anterior descending artery, *RCA* right coronary artery, *LCX* left circumflex artery, *LMT* left main trunk, *TIMI* thrombolysis in myocardial infarction, *BARC* Bleeding Academic Research Consortium.

*Persistent AKI vs. No AKI, P < 0.017 (Bonferroni correction). ^†^Transient AKI vs No AKI, P < 0.017 (Bonferroni correction).

Significant values are in bold.

### Long-term clinical outcomes

The median follow-up period was 1593 days (interquartile range, 903–2378 days), and the mean follow-up period was 1689 ± 926 years.

**Figure 2:**
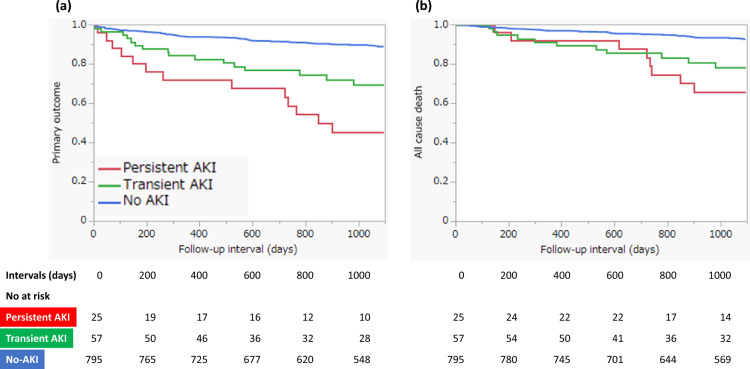
Kaplan–Meier survival curves of 3-year clinical outcomes. (**a**) Primary outcomes (death, myocardial infarction, hospitalization for heart failure, stroke, and initiation of maintenance dialysis); (**b**) All-cause death.

Figure 2 shows the Kaplan–Meier survival curves for clinical outcomes in the three groups. There was a significant difference in primary outcome-free survival (log-rank, P < 0.0001) and all-cause death-free survival (log-rank, P < 0.0001) among the three groups. In the pairwise post hoc tests, the cumulative incidence of the primary outcome was significantly higher in the persistent AKI (log-rank, P < 0.0001) and transient AKI (log-rank, P < 0.0001) groups than in the non-AKI group, and the cumulative incidence of all-cause death was significantly higher in the persistent AKI (log-rank, P < 0.0001) and transient AKI (log-rank, P = 0.0003) groups than in the non-AKI group. However, there was no significant difference between patients with persistent and transient AKIs in terms of the cumulative incidence of the primary outcome and all-cause death. Table 3 shows the incidence of primary and secondary outcomes in the three groups. Kaplan–Meier survival analysis also showed significant differences in MACEs, hospitalization for heart failure, stroke, and initiation of maintenance dialysis among the three groups. We investigated the predictors of primary outcomes using multivariate Cox proportional hazards analysis with two models. In both models, persistent AKI remained a significant predictor for primary outcome compared to non-AKI (model 1: HR, 2.68, 95% CI, 1.41–5.10, P = 0.0026; model 2: HR, 2.62, 95% CI, 1.38–4.99, P = 0.0034; Table 4). However, the incidence of transient AKI did not differ significantly from that of AKI in either model. Other predictors of primary outcomes in the multivariate analysis included age > 75 years; previous myocardial infarction, stroke, peripheral arterial disease (PAD); anemia; EF < 40%; and higher maximum CKMB level (Table 4).

**Table 3:** Long-term clinical outcomes.

	Persistent AKI	Transient AKI	No AKI	P value
Primary outcomes				
MACE + dialysis	13 (52.0)	16 (28.1)	84 (10.6)	**< 0.0001**
Secondary outcomes				
MACE	13 (52.0)	15 (26.3)	84 (10.6)	**< 0.0001**
All-cause death	8 (32.0)	11 (19.3)	53 (6.7)	**< 0.0001**
Myocardial infarction	1 (4.4)	1 (1.8)	10 (1.3)	0.45
Heart failure	4 (16.7)	2 (3.6)	38 (4.8)	**0.03**
Stroke	5 (20.8)	3 (5.4)	18 (2.3)	**< 0.0001**
Dialysis	0 (0)	2 (3.4)	1 (0.1)	**0.025**

Unless otherwise indicated, the values are presented as n (%). *AKI* acute kidney injury, *MACE* major adverse cardiovascular event.

Significant values are in bold.

**Table 4:** Univariate and multivariate Cox proportional hazards model for predictors of the primary outcomes.

	Univariate model			Multivariate model 1			Multivariate model 2		
	HR	95% CI	P value	HR	95% CI	P value	HR	95% CI	P value
Renal category									
No AKI	Reference	Reference	Reference	Reference	Reference	Reference	Reference	Reference	Reference
Transient AKI	3.07	1.80–5.24	**< 0.0001**	1.45	0.78–2.71	0.24	1.54	0.84–2.81	0.16
Persistent AKI	6.24	3.48–11.21	**< 0.0001**	2.68	1.41–5.10	**0.0026**	2.62	1.38–4.99	**0.0034**
Age > 75	3.36	2.32–4.87	**< 0.0001**	2.83	1.79–4.48	**< 0.0001**	2.76	1.76–4.33	**< 0.0001**
Male gender	0.75	0.50–1.14	**0.17**						
History of MI	2.34	1.31–4.16	**0.004**	2.19	1.20–4.02	**0.011**	2.16	1.18–3.96	**0.013**
Stroke or PAD	3.27	2.08–5.13	**< 0.0001**	2.55	1.55–4.18	**0.0002**	2.58	1.58–4.21	**0.0002**
Anemia	2.44	1.68–3.52	**< 0.0001**	1.67	1.06–2.64	**0.027**	1.71	1.08–2.69	**0.0213**
Bleeding BARC type ≥ 3	3.23	1.85–5.66	**< 0.0001**	1.66	0.87–3.18	0.13	1.63	0.81–3.29	0.17
eGFR	0.98	0.97–0.99	**< 0.0001**	1.00	0.99–1.01	0.97	1.00	0.99–1.01	0.93
EF < 40%	6.62	4.38–10.00	**< 0.0001**	2.98	1.82–4.87	**< 0.0001**	3.11	1.95–4.96	**< 0.0001**
Max CKMB	1.0011	1.0005–1.0017	**0.0003**	1.0016	1.0009–1.0023	**< 0.0001**	1.0016	1.0008–1.0022	**< 0.0001**
Killip ≥ 3	2.80	1.80–4.37	**< 0.0001**	1.50	0.90–2.50	0.12	1.45	0.86–2.44	0.16
Post PCI TMI score < 3	2.13	1.19–3.79	**0.0107**	1.37	0.73–2.56	0.33			
Mechanical circulatory support	1.78	1.13–2.78	**0.013**				1.02	0.58–1.79	0.95

*AKI* acute kidney injury, *CI* confidence interval, *EF* ejection fraction, *CK-MB* creatine kinase, *eGFR* estimated glomerular filtration rate, *MI* myocardial infarction, *PAD* peripheral artery disease, *BARC* Bleeding Academic Research Consortium, *PCI* percutaneous coronary intervention, *TIMI* thrombolysis in myocardial infarction.

Significant values are in bold.

### Predictors of AKI and persistent AKI

The predictors of AKI and persistent AKI evaluated using univariate logistic regression analyses are shown in Table 5. Age > 75 years, diabetes mellitus, anemia, bleeding with BARC type 3 or greater, Killip ≥ 3, low eGFR, EF < 40%, higher maximum CKMB level, femoral artery approach, multivessel PCI with one-time strategy, higher contrast volume/eGFR ratio, mechanical circulatory support use, and higher Mehran risk score were predictors of AKI. Age > 75 years, bleeding with BARC type 3 or greater, lower eGFR, EF < 40%, higher maximum CK-MB level, higher contrast volume/eGFR ratio, and a higher Mehran risk score were also predictors of persistent AKI.

**Table 5:** Univariate logistic regression for predictors of AKI and persistent AKI.

	Univariate model for AKI			Univariate model for persistent AKI		
	HR	95% CI	P value	HR	95% CI	P value
Male gender	0.82	0.49–1.38	0.46	0.51	0.22–1.17	0.11
Age > 75 years	2.52	1.60–3.96	**< 0.0001**	3.23	1.44–7.21	**0.0043**
Diabetes mellitus	2.00	1.28–3.14	**0.0025**	1.22	0.54–2.75	0.63
PAD	3.46	1.33–9.02	**0.011**	3.44	0.76–15.5	0.11
Anemia	3.11	1.98–4.90	**< 0.0001**	3.38	1.51–7.55	3.38
ARB, ACE-Is or ARNI	2.07	1.31–3.25	**0.0017**	1.80	0.81–4.03	0.15
Bleeding BARC type ≥ 3	5.19	2.62–10.27	**< 0.0001**	5.36	1.91–15.04	**0.006**
Killip ≥ 3	2.69	1.51–4.77	**0.0008**	2.22	0.81–6.07	0.12
eGFR	0.98	0.96–0.99	**< 0.0001**	0.977	0.958–0.997	**0.0203**
EF < 40%	4.43	2.46–7.99	**< 0.0001**	5.19	2.06–13.07	**0.0005**
Max CKMB	1.0008	1.0001–1.0017	**0.0473**	1.0014	1.0002–1.0027	**0.026**
Femoral artery approach	1.75	1.11–2.76	**0.017**	2.04	0.89–4.67	0.09
Multivessel PCI with one-time strategy	2.47	1.10–5.54	**0.029**	1.86	0.42–8.19	0.41
Contrast volume	0.998	0.994–1.001	0.22	0.997	0.990–1.004	0.37
Contrast volume/eGFR	1.42	1.21–1.66	**< 0.0001**	1.37	1.04–1.79	**0.023**
Mechanical circulatory support	1.77	1.01–3.10	**0.0451**	0.84	0.25–2.84	0.78
Mehran risk score	1.10	1.07–1.14	**< 0.0001**	1.060	1.001–1.121	**0.0444**

*AKI* acute kidney injury, *CI* confidence interval, *PAD* peripheral artery disease, *ARB* angiotensin II receptor blocker, *ACE-Is* angiotensin-converting enzyme inhibitors, *ARNI* angiotensin receptor neprilysin inhibitor, *BARC* Bleeding Academic Research Consortium, *EF* ejection fraction, *CK-MB* creatine kinase, *eGFR* estimated glomerular filtration rate, *MI* myocardial infarction, *TIMI* thrombolysis in myocardial infarction.

We investigated the predictors of persistent AKI using multivariate logistic regression analysis with four models containing three variables that were strongly relevant as predictors of persistent AKI in univariate analysis. Age > 75 years, EF < 40%, and higher maximum CK-MB level were independent predictors of persistent AKI in Model 1, age > 75 years and EF < 40% were independent predictors of persistent AKI in Models 2 and 3, and age > 75 years, EF < 40%, and bleeding with BARC type 3 or greater were independent predictors of persistent AKI in Model 4 (Table 6).

**Table 6:** Multivariate logistic regression analysis for predictors of persistent AKI.

	HR	95% CI	P value
Model 1			
Age > 75 years	3.45	1.43–8.37	**0.0006**
EF < 40%	3.43	1.31–9.01	**0.0124**
Max CKMB	1.0016	1.0002–1.0030	**0.0235**
Model 2			
Age > 75 years	3.02	1.24–7.31	**0.0146**
EF < 40%	4.13	1.51–11.26	**0.0056**
Contrast volume/eGFR	1.12	0.82–1.53	0.48
Model 3			
Age > 75 years	2.67	1.04–6.86	**0.04**
EF < 40%	4.08	1.49–11.17	**0.0061**
Mehran risk score	1.01	0.94–1.09	0.75
Model 4			
Age > 75 years	2.98	1.27–6.99	**0.0123**
EF < 40%	3.60	1.38–9.42	**0.0091**
Bleeding BARC type ≥ 3	3.74	1.14–12.29	**0.030**

*AKI* acute kidney injury, *CI* confidence interval, *EF* ejection fraction, *CK-MB* creatine kinase MB, *eGFR* estimated glomerular filtration rate, *BARC* Bleeding Academic Research Consortium.

Significant values are in bold.

## Discussion

The major findings of this study are that: (1) in patients who underwent emergency PCI for AMI, AKI was present in 82 (9.4%), and of the AKI, persistent AKI was present in 25 (30.5%) and transient AKI in 57 (69.5%); (2) primary outcome and all-cause death occurred more frequently in patients with persistent AKI and transient AKI than in those with non-AKI, and persistent AKI, but not transient AKI, was an independent predictor of primary outcome; (3) age > 75 years, EF < 40%, higher maximum CKMB level, and perioperative bleeding complication with BARC type 3 or greater were independent predictors of persistent AKI.

Contrast-induced nephropathy (CIN) is the main cause of renal dysfunction after PCI and is associated with increased long-term mortality and MACEs^12^. CIN is generally considered transient, with SCr levels typically reaching a peak within a few days and returning to baseline within 2 weeks in most cases^6^. However, some patients with CIN develop persistent increase in SCr levels.

Several studies have reported the incidence and prognostic impact of persistent and transient renal dysfunction after elective^13,14^ and emergency^15–18^ PCIs. The time interval for assessing persistent or transient renal dysfunction differed among studies. Some studies assessed persistent or transient renal dysfunction at short time intervals (2 weeks^17^ or at discharge^16–18^) from baseline, whereas others assessed long-term interval (1^15^, 3^13^, or 12 months^14^). Some patients, classified as having early persistent renal dysfunction, may have later improved their renal function. Therefore, we assessed persistent or transient renal dysfunction at long-term interval (1 month) from baseline. Despite the time intervals and definition for assessing persistent renal dysfunction among studies, the incidence of persistent renal dysfunction among patients with AKI was approximately 20–60%, which is similar to our result (30.5%).

In previous studies targeting patients who underwent elective PCI, Maioli et al.^13^ reported that both persistent and transient renal dysfunctions were independently associated with long-term mortality and MACEs, whereas Abe et al.^14^ reported that only persistent renal dysfunction was independently associated with increased long-term mortality. In previous studies targeting patients with AMI, Choi et al.^16^ reported that both persistent and transient renal dysfunction were independently associated with long-term mortality, whereas Kurogi et al.^17^ reported that persistent renal dysfunction, but not transient renal dysfunction, was independently associated with both long-term mortality and worse clinical outcomes. In the recent large-scale substudy^18^ from the MATRIX-Access (Minimizing Adverse Haemorrhagic Events by Transradial Access Site and Systemic Implementation of Angiox) trial, with a study population of 8201 patients who underwent catheter procedure for acute coronary syndrome (ACS), Landi et al. reported that in-hospital persistent but not transient AKI was independently associated with 1-year MACEs and mortality. The present study demonstrated that persistent renal dysfunction, but not transient renal dysfunction, was independently associated with poor long-term clinical outcomes. These studies, including the present one, consistently suggest that persistent renal dysfunction is associated with worse clinical outcomes. However, the effect of transient renal dysfunction on long-term clinical outcomes differs among studies. Nevertheless, the reversibility of renal dysfunction after AKI development has significant implications for the long-term follow-up of patients who undergo PCI.

Although several risk scores are available as predictors of CIN after cardiac catheterization procedures^19^, little is known about the predictors of persistent renal dysfunction. Some studies^20^ have investigated the predictors of persistent renal dysfunction and reported that the Mehran risk score^13,21^ and contrast volume/baseline eGFR ratio^17^ are useful for predicting persistent renal dysfunction. A recent study^22^ reported that the preprocedural N-terminal pro-B-type natriuretic peptide (NT-proBNP) level is useful for predicting persistent renal dysfunction. NT-proBNP reflects impaired cardiac output and increased inflammation^23^, which plays an important role in the development of persistent renal dysfunction. The present study demonstrated that EF < 40% and higher maximum CK-MB levels were strongly associated with the development of persistent renal dysfunction. Once AMI develops, cardiac function rapidly declines and cardiac damage is sustained. Subsequently, renal hypoperfusion following impaired cardiac output and systemic inflammatory response due to ischemic injury and myocardial necrosis may play important roles in the development of persistent renal dysfunction. Therefore, the assessment of cardiac function and the extent of myocardial necrosis after the onset of AMI might be useful for predicting the development of persistent renal dysfunction.

The present study demonstrated that major perioperative bleeding after PCI (BARC type 3 or greater) was not only associated with the development of AKI but also with the development of persistent renal dysfunction. A bleeding complication, especially one related to vascular access, is well known as a major complication after PCI. A previous study showed that bleeding complications after PCI are associated with the development of CIN^24^, the severity of which is closely correlated with the severity of bleeding. A sudden blood loss due to major bleeding such as BARC type 3 or greater may cause a serious impairment in renal perfusion, subsequently making AKI more severe and resulting in persistent renal dysfunction.

Early clinical follow-up, careful management, and close monitoring of renal function may improve long-term clinical outcomes in patients at high risk of developing persistent renal dysfunction after AKI. Additionally, the risk of bleeding complication is lower in PCI via the radial access than via a femoral access^25^. In high-risk patients of AKI, the choice of radial access may prevent the development of persistent renal dysfunction after PCI.

## Limitations

This study had several limitations. First, this was a single-center, retrospective observational study. Second, the high number of patients excluded due to the absence of analytical evaluation in the first month (approximately 9%). Third, the lack of data regarding patients who died during the index hospitalization (6.5%), specifically the time elapsed between PCI and death, as well as the progression of renal function in this subgroup. Forth, pharmacological treatments (diuretics, ACE-Is, ARB, and ARNI) and the examination using contrast media (contrast-enhanced computed tomography) after PCI, which might have influenced the worsening of renal function, were not included in the analysis. Fifth, we included only three variables to investigate the independent predictors of persistent AKI in the multivariate logistic regression analysis because of the small number of patients with persistent AKI. Sixth, the sample size was small and the present findings were considered exploratory in nature. Therefore, a large-scale prospective cohort study is required to verify our results. Seventh, the use of drugs such as ACE-Is, ARB, and ARNI, which improve prognosis after AMI, may be hindered by the presence of AKI and subsequent persistent renal dysfunction. As a result, its insufficient treatment may have worsened the prognosis in patients with persistent AKI. To clarify this causal relationship and identify the best therapeutic strategy in patients at high-risk of AKI, it is necessary to evaluate to what extent limitations in terms of the dosage of drugs potentially harmful to renal function in high-risk patients mitigate the progression to irreversible renal injury and influence the prognosis. A large-scale prospective study may therefore provide useful information for daily clinical practice.

To conclude, in patients who underwent emergency PCI for AMI, persistent AKI was independently associated with worse clinical outcomes, and advanced age, low cardiac function, greater myocardial necrosis, and perioperative major bleeding after PCI were predictors of persistent AKI.

## Data Availability

The datasets generated or analyzed during the current study are available from the corresponding author on reasonable request.

## References

[1] Tsai TT (2014). Contemporary incidence, predictors, and outcomes of acute kidney injury in patients undergoing percutaneous coronary interventions: Insights from the NCDR Cath-PCI registry. JACC Cardiovasc. Interv..

[2] Abe D (2014). Clinical predictors of contrast-induced acute kidney injury in patients undergoing emergency versus elective percutaneous coronary intervention. Circ. J..

[3] Narula A (2014). Contrast-induced acute kidney injury after primary percutaneous coronary intervention: Results from the HORIZONS-AMI substudy. Eur. Heart J..

[4] Sun G (2019). Contrast-induced nephropathy and long-term mortality after percutaneous coronary intervention in patients with acute myocardial infarction. Angiology.

[5] Shacham Y, Steinvil A, Arbel Y (2016). Acute kidney injury among ST elevation myocardial infarction patients treated by primary percutaneous coronary intervention: a multifactorial entity. J. Nephrol..

[6] Brown JR (2008). Transient and persistent renal dysfunction are predictors of survival after percutaneous coronary intervention: Insights from the Dartmouth Dynamic Registry. Catheter. Cardiovasc. Interv..

[7] Thygesen K (2012). Third universal definition of myocardial infarction. Circulation.

[8] Matsuo S (2009). Revised equations for estimated GFR from serum creatinine in Japan. Am. J. Kidney Dis..

[9] Mehran R (2011). Standardized bleeding definitions for cardiovascular clinical trials: A consensus report from the Bleeding Academic Research Consortium. Circulation.

[10] Mehta RL (2007). Acute Kidney Injury Network: Report of an initiative to improve outcomes in acute kidney injury. Crit. Care.

[11] Mehran R (2004). A simple risk score for prediction of contrast-induced nephropathy after percutaneous coronary intervention: Development and initial validation. J. Am. Coll. Cardiol..

[12] Rihal CS (2002). Incidence and prognostic importance of acute renal failure after percutaneous coronary intervention. Circulation.

[13] Maioli M (2012). Persistent renal damage after contrast-induced acute kidney injury: Incidence, evolution, risk factors, and prognosis. Circulation.

[14] Abe M (2017). Impact of transient or persistent contrast-induced nephropathy on long-term mortality after elective percutaneous coronary intervention. Am. J. Cardiol..

[15] Wi J (2011). Impact of contrast-induced acute kidney injury with transient or persistent renal dysfunction on long-term outcomes of patients with acute myocardial infarction undergoing percutaneous coronary intervention. Heart.

[16] Choi JS (2013). Relation between transient or persistent acute kidney injury and long-term mortality in patients with myocardial infarction. Am. J. Cardiol..

[17] Kurogi K (2019). Persistent renal dysfunction in patients undergoing primary percutaneous coronary intervention for acute myocardial infarction. J. Am. Heart Assoc..

[18] Landi A (2023). Transient vs in-hospital persistent acute kidney injury in patients with acute coronary syndrome. JACC Cardiovasc. Interv..

[19] Isaka Y (2019). Guideline on the use of iodinated contrast media in patients with kidney disease 2018. Circ. J..

[20] Watanabe M (2023). Prediction of persistent renal dysfunction following contrast-induced nephropathy after cardiac catheterization procedures. Circ. J..

[21] Wi J (2013). Prediction of contrast-induced nephropathy with persistent renal dysfunction and adverse long-term outcomes in patients with acute myocardial infarction using the Mehran risk score. Clin. Cardiol..

[22] Luo M (2023). Predictive value of N-terminal pro B-type natriuretic peptide for contrast-induced nephropathy non-recovery and poor outcomes among patients undergoing percutaneous coronary intervention. Circ. J..

[23] Jensen J (2010). Inflammation increases NT-proBNP and the NT-proBNP/BNP ratio. Clin. Res. Cardiol..

[24] Ohno Y (2013). Impact of periprocedural bleeding on incidence of contrast-induced acute kidney injury in patients treated with percutaneous coronary intervention. J. Am. Coll. Cardiol..

[25] Valgimigli M (2015). Radial versus femoral access in patients with acute coronary syndromes undergoing invasive management: A randomised multicentre trial. Lancet.

